# Machine Learning Methods in Predicting Patients with Suspected Myocardial Infarction Based on Short-Time HRV Data

**DOI:** 10.3390/s22187033

**Published:** 2022-09-17

**Authors:** Dmytro Chumachenko, Mykola Butkevych, Daniel Lode, Marcus Frohme, Kurt J. G. Schmailzl, Alina Nechyporenko

**Affiliations:** 1 Mathematical Modelling and Artificial Intelligence Department, National Aerospace University Kharkiv Aviation Institute, 61072 Kharkiv, Ukraine; 2 Molecular Biotechnology and Functional Genomics Department, Technical University of Applied Sciences Wildau, 15745 Wildau, Germany; 3 ccc. Center for Connected Health Care UG, 16818 Wustrau, Germany; 4 Systems Engineering Department, Kharkiv National University of Radio Electronics, 61166 Kharkiv, Ukraine

**Keywords:** myocardial infraction, heart rate variability, 10-second heart rate variability, diagnostics, machine learning, k-nearest neighbor classifier, radial basis function, decision tree, random forest

## Abstract

Diagnosis of cardiovascular diseases is an urgent task because they are the main cause of death for 32% of the world’s population. Particularly relevant are automated diagnostics using machine learning methods in the digitalization of healthcare and introduction of personalized medicine in healthcare institutions, including at the individual level when designing smart houses. Therefore, this study aims to analyze short 10-s electrocardiogram measurements taken from 12 leads. In addition, the task is to classify patients with suspected myocardial infarction using machine learning methods. We have developed four models based on the k-nearest neighbor classifier, radial basis function, decision tree, and random forest to do this. An analysis of time parameters showed that the most significant parameters for diagnosing myocardial infraction are SDNN, BPM, and IBI. An experimental investigation was conducted on the data of the open PTB-XL dataset for patients with suspected myocardial infarction. The results showed that, according to the parameters of the short ECG, it is possible to classify patients with a suspected myocardial infraction as sick and healthy with high accuracy. The optimized Random Forest model showed the best performance with an accuracy of 99.63%, and a root mean absolute error is less than 0.004. The proposed novel approach can be used for patients who do not have other indicators of heart attacks.

## 1. Introduction

Every year, information technology is becoming increasingly established in all areas of activity. Rapidly gaining momentum in recent decades and progress against the background of the widespread introduction of computer information technologies have also embraced medicine. The global COVID-19 pandemic has dramatically accelerated the pace of digitalization, causing entire industries to be transformed [1]. Today, information systems in medicine are used more and more widely: from making a diagnosis to forecasting the resources necessary for the continuous operation of medical institutions.

In healthcare, there are two groups of innovations—evolutionary [2] and revolutionary [3]. Evolutionary information technologies (IT) solutions help improve the quality of existing services: automate examinations, book patients online, and conduct screenings. Revolutionary ones are associated with new models of medical services, such as telemedicine or the use of artificial intelligence in diagnostics. A big driver of digital transformations in medicine is also a large accumulation of data: case histories, clinical analyses, etc. [4].

In addition, the impetus in healthcare informatization is the development of the artificial intelligence industry. Tools, powered by artificial intelligence (AI), uncover meaningful relationships in raw data. They can be applied to all areas of medicine, including drug discovery, medical diagnosis, treatment decision-making, patient care, and financial transactions and decisions. Artificial intelligence can make it easier to identify patterns by helping researchers create dynamic patient cohorts for research and clinical trials [5]. Modern machine learning tools that use artificial neural networks to learn highly complex relationships or deep learning technologies often outperform human capabilities in performing medical tasks. AI-enabled systems are capable of solving complex problems that are common in modern clinical care.

An analysis of modern research shows a growing prospect of using data-driven medical solutions for smart homes, which will turn the living environment into an innovative clinical environment for the prevention and early diagnosis of common diseases [6]. The introduction of personalized medicine solutions into the living environment will significantly reduce the risks of diseases associated with aging, including cardiovascular diseases [7].

Cardiovascular disease (CVD) is the leading cause of death worldwide: no other disease causes as many deaths yearly as CVD [8]. An estimated proportion of CVD among all global death is 32% [9]. Over 75% of CVD deaths occur in low- and middle-income countries [10]. This is mainly because people in low- and middle-income countries with CVD have less access to effective health care.

The primary behavioral risk factors for cardiovascular disease and stroke are unhealthy diet, lack of physical activity, tobacco use, and diabetes [11]. Such risk factors can manifest as high blood pressure, high blood glucose, high blood lipids, and being overweight and obese. Most cardiovascular diseases can be effectively managed not only by preventive measures but also by early diagnosis [12].

The global COVID-19 pandemic has become another challenge for health systems around the world in combating CVD, as they are one of the main complications of this infection after respiratory manifestations [13]. People with cardiovascular diseases are believed to be more susceptible to infection because the new coronavirus uses the Angiotensin-converting enzyme 2 (ACE2) receptor to enter the cell. People with cardiovascular complications during COVID-19 seem to have high levels of ACE2 expression, and SARS-CoV-2 uses it to dock onto the body’s cells to infect them [14].

The disastrous final route of coronary heart disease (CHD) in the world is myocardial infarction (MI) [15]. MI is damage to the heart muscle caused by an acute disruption of its blood supply due to blockage (thrombosis) of one of the heart’s arteries with atherosclerotic plaque [16]. In this case, the affected cardiac muscle cells die by necrosis, and in the further course the necrotic district changes into a fibrous scar. Cell death begins within 20–40 min from the moment of cessation of blood flow in the coronary artery. The high prevalence and narrow time window demand new methods for diagnosing early-stage MI to prevent patient lethality. Therefore, developing models and methods for the early diagnosis of MI is an urgent task. This shall reduce the mortality rate from MI and open up access for countries and people who do not have special equipment and enough medical personnel to prevent MI. The models proposed in this study are based on statistical machine learning methods and do not require high computational power and special equipment. The use of the proposed models is possible on personal computers.

In order for a patient to be diagnosed with myocardial infarction, they must fulfill at least two of the following three criteria, according to the World Health Organization:

- Clinical history of chest discomfort consistent with ischemia, such as crushing chest pain;
- An elevation of cardiac markers in the blood (Troponin-I, CK-MB, Myoglobin);
- Characteristic changes on electrocardiographic tracings taken serially.

The significant electrocardiography (ECG) changes indicative of myocardial infarction are the elevation (in STEMI) or depression (NSTEMI) of the ST segment, as well as the inversion of the T wave (in NSTEMI) [17]. However, this requires the patient to visit the emergency department of a hospital or a doctor’s office, which necessarily means some delay in diagnosis and treatment. A very easy and “at home” applicable ECG-based heart attack diagnosis would be desirable.

Given this, there is a crucial need to study new signs that have diagnostic value for the diagnosis of myocardial infarction. In this paper, we will study the impact of heart rate variability (HRV) time domain metrics, which are obtained from short 10-second samples of ECG. One more aim of the current research is to develop an effective machine learning model for MI diagnostics.

To achieve this aim, the following requirements need to be met:

- Current research on MI classification should be analyzed;
- Data should be analyzed;
- A machine learning models should be developed;
- Data should be prepared for the experimental study with developed machine learning models;
- Experimental evaluation of the developed models with open data on MI should be provided;
- Comparative analysis of obtained results with other methods and models should be provided.

The respective contribution of this study is two-fold—Firstly, the development of machine learning models based on a k-nearest neighbor classifier, radial basis function, decision tree, and random forest will allow for estimating accuracy of simple machine learning techniques for cardiovascular diseases classification; Secondly, the study of the diagnostic value of data, HRV, obtained from the original ECG signal, as an alternative to such characteristic changes in the ECG curve as ST-segment elevation or depression, T-wave inversion, confirming the diagnosis of MI. Moreover, research on HRV metrics is particularly interesting as they are derived from short 10-second ECG records.

The rest of this publication is structured as follows: Section 2 provides the current state of cardiovascular disease machine learning classification models and methods. Section 3, namely Materials and Methods, describes machine learning models based on k-nearest neighbors classifier, radial basis function, decision tree, and random forest methods. Section 4 provides analysis of the publicly available dataset PTB-XL and results of data preprocessing and preparation. Section 5 provides the results of experiments with developed models and the results of model optimization. Section 6 presents the conclusions and future work.

Given research is part of a complex intelligent information system for epidemiological diagnostics, developed within the project 2020.02/0404 “Development of intelligent technologies for assessing the epidemic situation to support decision-making within the population biosafety management” funded by National Research Foundation of Ukraine, the concept of which is discussed in [18].

## 2. Current State of Research

The most effective method for automated diagnosis of myocardial infarction is the analysis of electrocardiogram data. ECG is a method for analyzing the work of the heart based on the registration of electromagnetic field variations that occur in the heart muscle during the cardiac cycle [1]. The signal that reflects the nature of these variations is called an electro-cardio signal (ECS). Analysis of the ECS is a process of studying the ECG signal, aimed at detecting pathological abnormalities in its individual sections and determining the causes of these abnormalities.

The main problems that arise during the analysis of the ECG can be classified by reason of their occurrence into [19]:

- stochastic nature of the biological system under study;
- imperfection of the technical means of signal pickup.
  There are three main stages in the task of automated ECG analysis [20,21]:
- pre-processing stage, in which the signal is separated from interference;
- conversion stage, at which informative features of the signal are extracted;
- the stage of solving the problem, which generates the output signal according to the identified informative features.

The task of ECG classification is to identify informative signs and find their dependence on the corresponding heart disease or its absence. To date, the methods based on neural networks show the highest accuracy among the methods of automated CVD diagnostics. However, their disadvantage is the high computational complexity and the need for computing resources [22]. This is not feasible in the context of health care facilities in low- and middle-income countries, which account for most deaths. Therefore, machine learning methods not based on artificial neural networks are preferred in this study.

Authors of Ref. [23] have built classification models of MI using 192 lead body surface potential maps analysis. The most important features were used as input to a series of supervised classification models using Naive Bayes, Support Vector Machine, and Random Forest methods. The accuracy of the constructed models was 81.9% for Naive Bayes, 82.8% for Support Vector Machine, and 84.5% for Random Forest. However, using 192 leads for the detection is not practical for the detection of MI.

Polat et al. [24] have modified the k-nearest neighbors method and used it as a preprocessing approach before the classification. Artificial immune recognition system with a fuzzy resource allocation mechanism as a classifier, showed an accuracy of 87.0% for MI diagnosing. In Ref. [25], the decision tree and bagging based on decision tree models are proposed. The authors have used the database of 920 samples. The accuracy is 78.91% for decision tree and 81.41% for bagging. Ref. [26] proposes the modification of the decision tree method by nine voting equal frequency discretization gain ratio. Based on the data from 297 samples, authors obtained an accuracy of 84.1%.

Ref. [27] discussed two machine learning approaches to ECG classification. Models based on support vector machine and radial basis function network have shown accuracy 85.05% and 82.71%, respectively, using 5-fold cross-validation, and 85.05% and 82.24% using 10-fold cross-validation.

In Ref. [28], the ensemble method for heart diseases classification is proposed, which integrates k-means clustering with naïve Bayes. The best accuracy obtained for two clusters random row initial centroid selection is 84.5%. Chitra and Seenivasagam [29], to validate the developed cascaded neural network of CVD classification, have built the model based on a support vector machine and obtained an accuracy of 77.5% with it. Authors of [30] have proposed three machine learning models of heart disease detection. The accuracy obtained with gain ratio decision tree is 79.1%, the accuracy of Naïve Bayes method is 83.5%, and the accuracy of k-nearest neighbor method with K = 19 is 83.2%.

Authors of [31] used data from 143 cases, 79 of which were MI-related. The machine learning model based on k-nearest neighbors method showed an accuracy of 87.0% with K = 4. Authors of [32] have used the weighted vote-based ensemble technique to combine the results of Naive Bayes, decision tree based on information gain, decision tree based on Gini index, instance-based learner, and support vector machine algorithms. The accuracy of obtained ensemble model is 87.37%. Authors of [33] modified the proposed in the Ref. [26] method using nine voting equal frequency discretization with Gini index decision tree applied to the same dataset and obtained an accuracy of 85.3%.

In Ref. [34], ECG data taken for six seconds and ECG data taken the entire length of the data in two minutes are investigated. The developed model of modified K-nearest neighbors showed an accuracy of 71.2% with K = 3.

The comparative analysis of investigated researches is presented in Table 1.

Ref. [35] analyzes recent research on classifying HRV data using machine learning models. However, most of them are focused on stress classification. In addition, this paper discusses the features of various durations of HRV records and metrics in the time and frequency domains in the context of diagnosing cardiovascular diseases. Thus, studying the effect of HRV indicators obtained on the basis of short 10-second ECG recordings on the accuracy of myocardial infarction classification based on machine learning models is of particular research interest.

The study of [36] investigated 10-second heart rate variability. The authors concluded that using the 10-second estimate would be of extreme benefit in assessing HRV as opposed to a need to record a 24-h ECG. However, no further investigations of its features have been provided.

Analysis of the current state of research on ECG processing and diagnosing MI using machine learning models also shows that data preprocessing is a crucial step to achieving a high degree of accuracy in the training model. Heterogeneous data also play a vital role in the accuracy of classifiers. Reviewed studies say that machine learning classifiers with preprocessed data show more accurate results than those without preprocessed data.

## 3. Materials and Methods

Within research, four models based on machine learning methods for the classification of patients with MI were developed. Machine learning models are based on k-nearest neighbors classifier, radial basis function, decision tree, and random forest.

### 3.1. K-Nearest Neighbor Classifier

The principle behind k-nearest neighbor method is to find a predetermined number of training samples closest in the distance to a new point and provide a value for the data [37]. Despite its simplicity, the k-nearest neighbor method has succeeded in many classification and regression problems, including the medical domain. Being a non-parametric method, it is often successful in classification situations where the decision limit is unclear.

Euclidean distance is a commonly used distance metric for continuous variables [38]. For discrete variables, such as text classification, you can use another metric, such as the overlap metric (or Hamming distance) [39]. In addition, k-nearest neighbors classifier is used with correlation coefficients such as Pearson and Spearman [40]. Often, classification accuracy can be greatly improved if the distance metric is learned using specialized algorithms, such as high-margin, nearest-neighbor, or neighborhood component analysis.

The disadvantage of the primary majority vote classification is that the class distribution is skewed. More frequent class examples tend to dominate the prediction of a new example since they tend to be spread among nearest neighbors due to their large number.

One way to overcome this problem is to weight the classification given the distance from the control point to each of its nearest neighbors. The class (or value in regression problems) of each of the k closest points is multiplied by a weight proportional to the reciprocal distance from that point to the control point. Another way to overcome skew is to abstract the data representation.

To classify the objects of the test sample, you must sequentially perform the following steps:

1. To calculate the distance to each of the objects in the training sample;
2. To select the object of the training sample, the distance to which is minimal;
3. The class of the classified object is the class that occurs among k nearest neighbors most often.

Euclidean distance in multidimensional feature space is calculated as follows:

(1) $$d_{ab}=\sqrt{\sum_{i=1}^{n}{\left(x_{ai}-x_{bi}\right)}^{2}},$$

where *a* and *b* are points in *n*-dimensional space;

*i* is ordinal number of the feature;

*x_ai_* and *x_bi_* are coordinates of points *a* and *b* by the feature *i*.

The class with the most votes is assigned to the new element:

(2) $$y_{a}\left(a,X,k\right)=argmax_{y\in Y}\sum_{i=1}^{k}\left(y_{a}^{i}=y\right),$$

where *a* is a new element (connection),

*X* is a training sample,

*y* is a class,

*Y* is a set of classes,

*y_a_^i^* is the class of *i*-th neighbor *a*,

*k* is the number of neighbors.

### 3.2. Radial Basis Function

In machine learning, a radial basis function is used in various kernel learning algorithms [41]. In particular, it is commonly used to classify support vector machines. The radial basis function kernel on two samples *x* and *x′*, represented as feature vectors in some input space, is defined as:

(3) $$K\left(x,x^{\prime }\right)=exp\left(\frac{{-∥x-x^{\prime }∥}^{2}}{2\sigma^{2}}\right),$$

where ${∥x-x^{\prime }∥}^{2}$ can be defined as the square of the Euclidean distance between two feature vectors,

*σ* is free parameter.

The equivalent definition includes the parameter $\gamma =\frac{1}{2\sigma^{2}}$:

(4) $$K\left(x,x^{\prime }\right)=exp({-\gamma ∥x-x^{\prime }∥}^{2}).\;$$

Since the value of the RBF kernel decreases with distance and ranges from zero (at the boundary) to one (when *x = x′*), it has a ready interpretation as a measure of similarity [42]. The feature space of the kernel has an infinite number of dimensions; for *σ =* 1, it grows:

(5) $$exp\left(\frac{-1}{2}{∥x-x^{\prime }∥}^{2}\right)=exp\left(\frac{2}{2}x^{T}x^{\prime }-\frac{1}{2}{∥x∥}^{2}-\frac{1}{2}{∥x^{\prime }∥}^{2}\right)=\;exp\left(x^{T}x^{\prime }\right)exp\left(\frac{-1}{2}{∥x∥}^{2}\right)exp\left(\frac{-1}{2}{∥x^{\prime }∥}^{2}\right)=\;\sum_{j=0}^{\infty }\frac{\left(x^{T}x^{\prime }\right)}{j!}exp\left(\frac{-1}{2}{∥x∥}^{2}\right)exp\left(\frac{-1}{2}{∥x^{\prime }∥}^{2}\right)=\;\sum_{j=0}^{\infty }\sum_{\sum n_{i}=j}\frac{\left(x^{T}x^{\prime }\right)}{j!}exp\left(\frac{-1}{2}{∥x∥}^{2}\right)exp\left(\frac{-1}{2}{∥x^{\prime }∥}^{2}\right).$$

### 3.3. Decision Tree

Decision Trees is a non-parametric supervised learning technique used for classification and regression [43]. The goal is to create a model that predicts the value of the target variable by learning simple decision rules derived from the characteristics of the data. The tree can be considered as a piecewise constant approximation.

Decision trees are trained on the data to approximate a sinusoid using a set of if-then-else decision rules. The deeper the tree, the more complex the decision rules and the better the model. The benefits of decision trees include:

- Easy to understand and interpret;
- Trees can be visualized;
- Requires little data preparation;
- Tree usage weights are the logarithmic number of data points used to train the tree.
  The disadvantages of decision trees include:
- Trained decision models can create highly complex trees that do not generalize well. To avoid this problem, mechanisms such as pruning, setting a minimum number of samples required in a leaf node, or setting a maximum tree depth is needed.
- Decision trees can be unstable because minor variations in the data can result in a completely different tree. The use of ensemble decision trees mitigates this problem.

The problem of learning an optimal decision tree is NP-complete in several aspects of optimum, even for simple concepts. Therefore, practical decision tree learning algorithms are based on heuristic algorithms such as the greedy algorithm, where locally optimal decisions are made at each node. Such algorithms cannot guarantee the return of a globally optimal decision tree. This can be mitigated by training multiple trees in an ensemble where features and samples are randomly sampled with replacement.

### 3.4. Random Forest

Random forest is a type of supervised machine learning algorithm based on ensemble learning [44]. Ensemble learning is a type of learning where you combine different types of algorithms or the same algorithm multiple times to form a more powerful prediction model. The random forest algorithm combines several algorithms of the same type, that is, several decision trees, resulting in a forest of trees, hence the name Random Forest. The random forest algorithm can be used for both regression and classification problems.

These two sources of randomness aim to reduce the variance of the forest estimate. Individual decision trees typically exhibit high variance and tend to overflow. The introduced randomness in forests yields decision trees with slightly isolated prediction errors. By taking the average of these predictions, some errors can be avoided. Random forests achieve reduced variance by combining diverse trees, sometimes at the cost of a slight increase in bias. In practice, the reduction in variance is often significant, giving an overall better model.

Benefits of using Random Forest include:

- The random forest algorithm is not biased because there are multiple trees, and each tree learns from a subset of the data. Basically, the random forest algorithm relies on the power of the “crowd”; therefore, the overall bias of the algorithm is reduced.
- The algorithm is stable. Even if a new data point is introduced into the data set, the overall algorithm is not significantly affected because the new data may affect one tree. However, it is challenging to affect all trees.
- The random forest algorithm works well if the sample contains both categorical and numerical features. The random forest algorithm also performs well when data are missing or have not been well scaled.

The main disadvantage of random forest is its complexity. The model takes out much more computational resources due to a large number of merged decision trees. Due to their complexity, they take much longer to train other similar algorithms.

## 4. Data Analysis and Preprocessing

For the experimental study, we used the open database PTB-XL [45], which was collected within the project PhysioNet [46]. PTB-XL is the to-date largest freely accessible clinical 12-lead ECG-waveform dataset comprising 21,837 records from 18,885 patients of 10 seconds length [47]. Two cardiologists annotate the ECG data as a multi-label dataset, where the diagnostic labels have been further grouped into superclasses and subclasses. The data set spans many diagnostic classes, including healthy individuals. The data also contain demographic metadata, additional diagnostic statements, and probabilities of diagnosis, which are manually annotated.

### 4.1. Input Data Description

**Figure 1:**
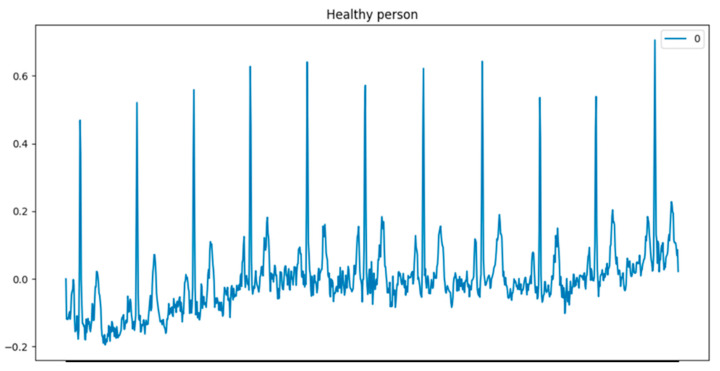
Example of ECG data.

The initial dataset consists of 15,014 records, 9528 normal, and 5486 myocardial infarction (MI) data, including ECG signals and metadata. The random patient examination ECG data is shown in Figure 1. One can single out a clear cyclically repeating pattern with some modifications, and a baseline drift, which must be eliminated using signal preprocessing techniques. The patient dataset also contains the corresponding metadata.

**Figure 2:**
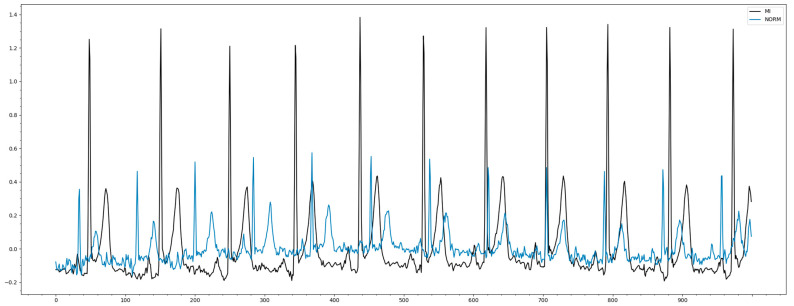
Example of ECG data (Norm and MI).

The metadata can be divided into the following categories:

1. Identifiers: Each entry is identified by a unique ecg_id. The eligible patient is encoded via the patient ID; paths to the original recording (500 Hz) and the downsampled version of the recording (100 Hz);
2. General metadata: demographic and registration metadata such as age, gender, height, weight, nurse, site, device, and date of enrollment;
3. ECG operations: The main components are scp_codes (SCP-ECG operations as a dictionary with entries of the form statement: probability, where probability is set to 0 if unknown) and report (report string). Additional fields are heart_axis, infarction_stadium1, infarction_stadium2, validated_by, second_opinion, initial_autogenerated_report, and validated_by_human;
4. Signal metadata: signal quality such as noise (static_noise and burst_noise), baseline offset (baseline_drift), and other parameters such as electrodes_problems;
5. Figure 2 shows records of two random patients: normal and with MI.

**Figure 3:**
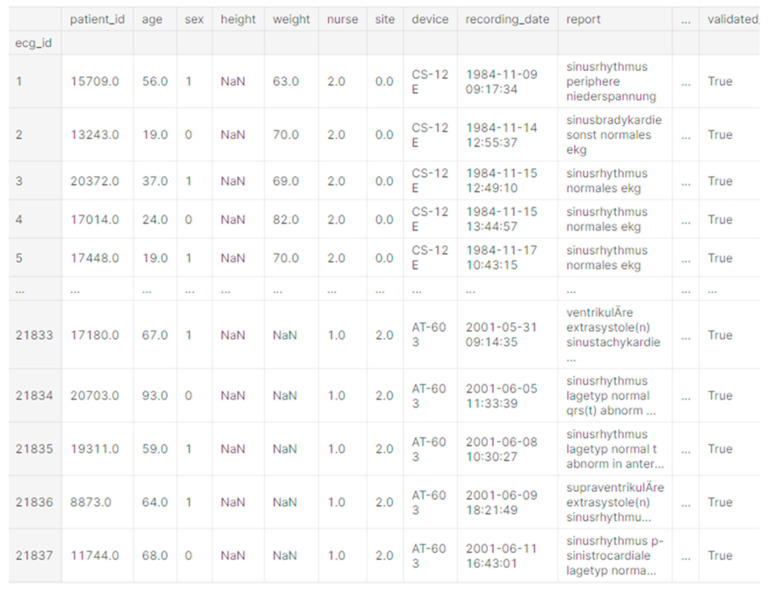
Primary dataset review.

Figure 3 shows the basic data about the patient and information about the examination, biological data, and diagnosis.

### 4.2. ECG Data Analysis

Based on studies [48] regarding the diagnostic value of HRV indicators for the detection of cardiovascular diseases and, in particular, myocardial infarction, ECG signals were processed as follows: First, Heart Rate Variability (HRV) data were obtained from the original ECG signal, and then the time-domain metrics were calculated. HRV is the fluctuation in the time intervals between adjacent heartbeats that correspond to R peaks on the ECG signal. The time-domain metrics include inter-beat interval (IBI), heartbeats per minute (BPM), the standard deviation of the R peak to R peak intervals (SDNN), with the assumption that the data only come from the Normal to Normal sinus rhythm and the root mean square of successive differences (RMSSD). RMSSD is determined by first calculating each successive R peak to R peak. Afterward, each of these values is squared, and the results are averaged before finding the square root of the total. Equations (6) and (7) describe the SDNN and RMSSD calculations, respectively:

(6) $$\mathrm{SDNN}=\sqrt{\frac{1}{N-1}\overset{N}{\underset{i=1}{\sum }}{\left(X_{i}-\mu \right)}^{2}}$$

(7) $$\mathrm{RMSSD}=\sqrt{\frac{1}{N}\overset{N-1}{\underset{i=1}{\sum }}{\left(X_{i}-X_{i+1}\right)}^{2}}$$

**Figure 4:**
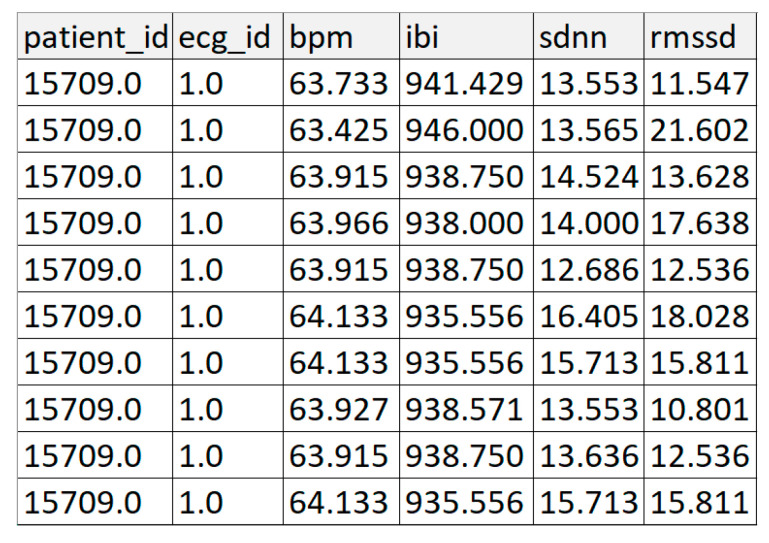
Results of ECG signal processing.

The extracted features for analysis are presented in Figure 4.

### 4.3. Data Preprocessing

Both ECG signal data and metadata comprise noises, missing values, and other inconsistencies and require preprocessing. For these purposes, noise reduction techniques were applied to ECG signals. It allowed us to enhance ECG peaks, convolving synthetic QRS templates with the signal, and applying a notch filter, resulting in a strong signal-to-noise ratio.

The resulting disease classes were divided into negative and positive diagnosis values of 0 and 1, respectively. The MI class was classified as positive and assigned a value of 1, and the NORM class was classified as negative and assigned a value of 0.

**Figure 5:**
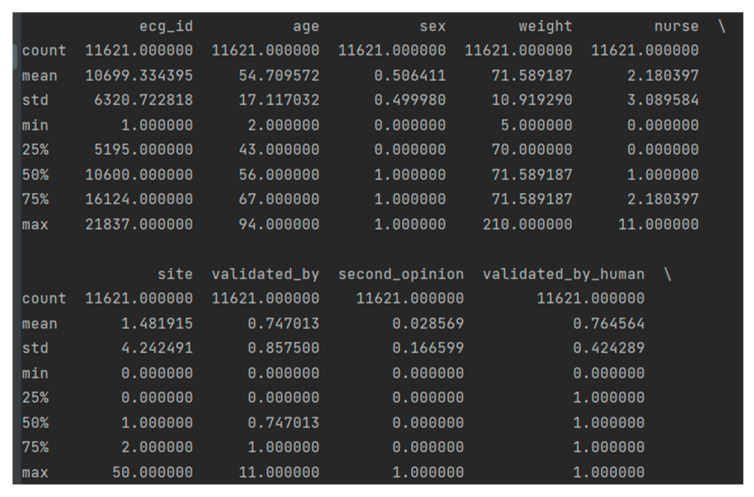
Data sample descriptive statistics.

The descriptive statistics shown in Figure 5 include statistics summarizing the major trend, variance, and distribution shape of the data set, excluding NaN values. The average age of patients is quite high with 52 years, and the 50% percentile is 54 years. It can be seen that half of the resulting sample are men and the other half are women. The majority of the 76% sample was re-validated by another physician. In addition, 10 ECG machines and 11 nurses participated in the process, which could affect the result.

**Figure 6:**
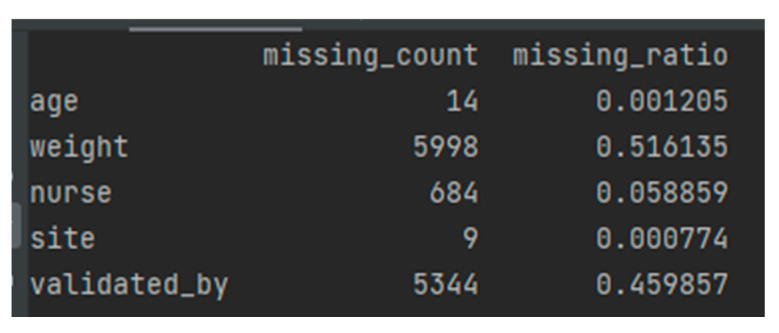
Missed data in the obtained data sample.

When data values for a variable in observation are not stored, they are missing data or missing values. Missing data are common and can have a significant impact on the conclusions that can be drawn from the data. Figure 6 shows a list of missing values in the analyzed dataset.

Most cases of missing values are noted in the excess mortality columns, which will not be used for predictions or testing but only for analysis. They can be easily dropped. Other values seem to be missing because there were no specific observations or studies on certain days.

The following methods can be applied to fill in the missing values:

- Linear interpolation;
- Linear interpolation of neighboring values.

In our case, since all records are not related to each other, interpolation options are not suitable since, in this case, there is no task to preserve the original behavior. 

Missing values can be filled in with:

- Parameters Outlier or Zero;
- Mean value;
- Median;
- Constant.

Filling missing values with a constant or zero is not sufficient and not a good option. Filling in missing values with the last value may give better results when using the mean or median. The median method was chosen because it has an advantage over the mean values in a situation where some values are anomalous and strongly bias the mean.

In order to calculate the correlation of the Pearson product with a moment, one first needs to determine the covariance of the two variables in question. Next, you need to calculate the standard deviation of each variable. The correlation coefficient is determined by dividing the covariance by the product of the standard deviations of the two variables. Based on the analysis of medical data, the following conclusions can be drawn:

- Age has an average negative correlation with diagnosis;
- Gender has a medium negative correlation with weight and a low positive correlation with diagnosis.

Both significantly influence the diagnosis parameters infraction stadium and heart axis.

Medical parameters have an average positive correlation with the diagnosis. The heart axis indicates the heart’s position relative to the body and its inclination [49]. Abnormal values may indicate related diseases. In turn, infraction stadium is a disease in which the patient has a brain tumor–glioma [50].

## 5. Results

After preliminary analysis and data preparation, we obtained 13,480 records, and separate our data into training and validation data. We want to provide the model with as much training data as possible. However, we also want to ensure we have enough data to test the model. As the number of rows in the dataset increases, we can provide more data to the training set. Another critical parameter is data mixing.

In this study, the set was distributed in the ratio of 80% to 20% for training and validation data. A set of regression classifiers and machine learning models was defined for testing with this data set. Tests of statistical significance were carried out to check the validity of the result [51]. To do this, we evaluated the model 10 times and obtained the average values of accuracy and RMSE.

The results of the developed machine learning models are shown in Table 2.

It can be concluded that, with a high probability, the Random Forest classifier gives the best results. The results shown are competitive with those presented in Table 1. To improve accuracy, it is necessary to select parameters to optimize the results obtained for classification by the Random Forest model.

The Random Forest classifier is trained using load aggregation, where each new tree is selected from a sample of load observations. Out of bag is the average error for each computed using tree predictions not contained in the corresponding load sample. This allows the built Random Forest model to fit and validate during training.

The main parameters to be adjusted when using these methods are n_estimators and max_features. The first is the number of trees in the forest. The more, the better, but also the more time it will take to calculate. In addition, the results will no longer improve significantly over a critical number of trees. The latter is the size of random feature subsets that should be considered when splitting a node. The more minor, the more significant the reduction in dispersion, but also the more significant the increase in bias. The empirically “correct” defaults are: max_features = None (always consider all features instead of a random subset) for regression problems and max_features = “sqrt” (using a random subset of size sqrt(n_features)) for classification problems (where n_features is the number of features in the data). Good results are often achieved by setting max_depth = None in combination with min_samples_split = 2 (i.e., when trees are fully developed). However, these values are usually not optimal and can result in models that consume a lot of RAM.

The best parameter values should be cross-validated. Cross-validation technology is the most common using the most common K-Fold CV method. Typically, the data are divided into training and validation. However, for the K-Fold technique, the training data are split into K different samples, which are called Fold. By iteratively selecting samples, the model accuracy result is evaluated. After that, the average performance is found, which is the validation metric.

In the case of RandomForest, the following can be controlled as parameters:

- The number of trees in the forest (n_estimators);
- The maximum depth of the tree (max_depth). If not, then the nodes are expanded until all leaves are clean or until all leaves contain less than min_samples_split samples;
- The minimum number of samples (min_samples) that must be in a leaf node. A split point at any depth will only be considered if it leaves at least min_samples_leaf training samples in each of the left and right branches. This can have the effect of smoothing the model.

In addition, having selected the optimal hyperparameters, we can determine the maximum number of max_features features used to find the best test data split ratio:

For “Sqrt” parameter:

(8) $$max_{features}=\sqrt{n_{features}}.\;$$

For “Log2” parameter:

(9) $$max_{features}=logn_{features}.\;$$

For “None” parameter:

(10) $$max_{features}=n_{features}.\;$$

**Figure 7:**
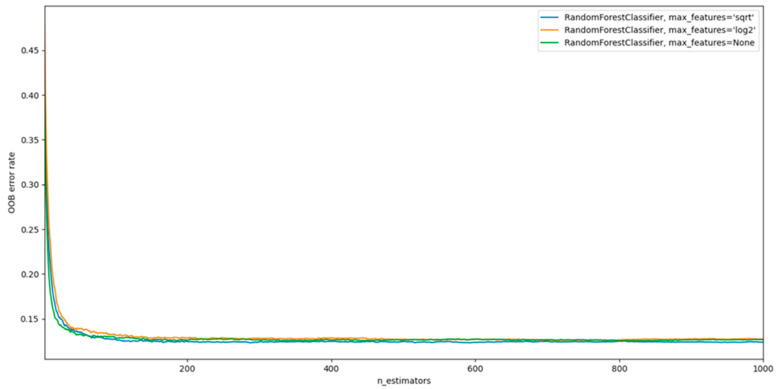
Results of Random Forest model with different parameters.

The results are presented in Figure 7.

Analyzing Figure 6, we can conclude that the best strategy for choosing max_features is “None” (all features are always considered instead of a random subset). However, this configuration requires more system resources. In addition, the critical number of trees (n_estimators) is around 300.

**Figure 8:**
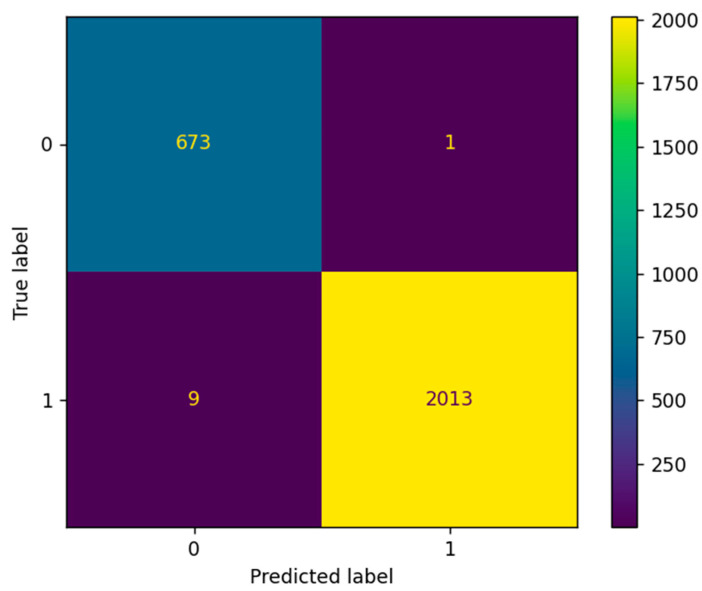
Correspondence matrix of the provided and actual values of the constructed model.

Figure 8 shows the matrix of correspondences between the provided and actual values of the constructed model.

The accuracy and absolute error of the data are checked against the validation data. The building and training of the model were carried out several times, and the accuracy was maintained at 99.629 %, which is 2% better than the first model tuning. Even when mixing data at the stages of data preparation, they do not affect the result in any way. The resulting stability can be explained by the properties of the tree structure of the algorithm.

The accuracy of the optimized model is 99.629%, and root mean absolute error is 0.0037.

## 6. Conclusions

In existing studies in patients with myocardial infarction, the HRV time domain indicators are mainly isolated from long-term measurements from 5 min to 24 h. On short measurements, 10 s, the time parameters were not studied properly. However, this is an essential task because the protocol for examining patients with suspected MI says the patient should undergo an ECG for 10 s with 12 leads. In addition, this leads to the conclusion that HRV parameters have potential clinical value in cases where analysis of ECG data did not reveal changes in signal morphology. The evidence for correlations between changes in HRV parameters and ST-segment changes in the classic 12-lead ECG is still limited. Although the number of cases is still small and the results are based on ECG databases (and not on real-world clinical data), there are encouraging results on this and we are working on it ourselves.

An analysis of time parameters showed that the most significant parameters for diagnosing MI are sdnn, bpm, and ibi.

As part of this study, an experimental study was conducted on the data of the open PTB-XL dataset for patients with suspected MI. The results showed that, according to the parameters of the 10-second ECG, it is possible to classify patients with suspected MI as sick and healthy. Four machine learning methods were analyzed: k-nearest neighbors classifier, radial basis function, decision tree, and random forest. All methods showed high accuracy. However, the optimized Random Forest method showed an accuracy of 99.629%.

Unfortunately, we all know cases in which one or more pieces of the mosaic for the diagnosis of myocardial infarction ultimately proved to be false positives or negatives, and this is one reason for the need to be able to look at all the “big” pieces of the mosaic as far as possible: a typical clinical picture, typical ECG changes, and a troponin increase. For troponin, there are point-of-care tests, i.e., rapid tests made near the patient (they are not perfectly specific compared to the “normal” laboratory tests, but they allow very often a more in-depth assessment of the case). For ECG diagnostics, we believe that a possibility should be created to obtain the necessary information early and, if possible, already “at home” and by the patient himself or his relatives. Furthermore, for this, in our opinion, a classic 12-lead ECG is not suitable, but perhaps a wearable patch that allows HRV analysis. The analysis should be supported by AI. In addition, even if this information from HRV is also not 100% sensitive and specific, it seems plausible to combine it with a rapid troponin test. The affected patient who has acute chest pain could perform both at home, and an AI algorithm could derive a recommendation for action from the information now available (typical symptomatology yes/no, HRV suspicious of myocardial infarction yes/no, troponin elevated yes/no): Alert and transport to emergency department or visit primary care physician’s office at the earliest possible date.

The proposed approach can be used for patients who do not have other indicators of heart attacks.

In the future, it is planned to conduct studies on each individual leading to determine the minimum number of leads required to obtain reliable results for the diagnosis of MI. This will allow the proposed methodology to be applied outside medical institutions and integrated into the smart home system. The proposed automated solution based on machine learning models is a practical addition to traditional diagnostic approaches and saves resources while supporting decision-making by doctors. This is especially important in the context of the global COVID-19 pandemic when healthcare resources are limited and patients do not always have access to their family doctors regularly.

## Figures and Tables

**Table 1:** Comparison of accuracy of current researches.

Author, Source	Approach	Accuracy
Yuwono T., et al. [34]	Modified K-nearestneighbors with K = 3	71.2%
Chitra R., Seenivasagam V. [32]	Support vector machine	77.5%
Tu M.C., et al. [25]	Decision tree	78.91%
Shouman M., et al. [30]	Gain ratio decision tree	79.1%
Tu M.C., et al. [25]	Bagging based on decision tree	81.41%
Zheng H., et al. [23]	Naïve Bayes	81.9%
Ghumbre S., et al. [27]	Radial basis function network using 10-fold cross-validation	82.24%
Ghumbre S., et al. [27]	Radial basis function network using 5-fold cross-validation	82.71%
Zheng H., et al. [23]	Support vector machine	82.8%
Shouman M., et al. [30]	K-nearest neighbors with K = 19	83.2%
Shouman M., et al. [30]	Naïve Bayes	83.5%
Shouman M., et al. [26]	Equal frequency discretization gain ratio decision tree	84.1%
Zheng H., et al. [23]	Random forest	84.5%
Shouman M., et al. [28]	Ensemble: k-means with Naïve Bayes	84.5%
Ghumbre S., et al. [27]	Support vector machine	85.05%
Kirmani M.M., et al. [33]	Nine voting equal frequency discretization with Gini index decision tree	85.3%
Polat K., et al. [24]	Artificial immune recognition system	87.0%
Yuwono T., et al. [31]	K-nearest neighbor	87.0%
Bashir S., et al. [32]	Ensemble: Naive Bayes, decision tree based on information gain, decision tree based on Gini index, instance-based learner, support vector machine	87.37%

**Table 2:** Results of simulation.

Machine Learning Model	Accuracy	Root Mean Square Error
K-nearest neighbors classifier	71.105%	0.289
Radial basis function	75.408%	0.245
Decision tree	89.867%	0.109
Random forest	97.774%	0.022

## Data Availability

The initial data used in this research are publicly available by the link https://physionet.org/content/ptb-xl/1.0.2/ (accessed on 21 August 2022).
